# Effects of Enzymatic Hydrolysis Time on the Structure and Properties of Chickpea Porous Starch and Its Performance as a Curcumin Carrier

**DOI:** 10.3390/foods15213895

**Published:** 2026-10-30

**Authors:** Yuqing Wang, Liwen Chai, Xudong Che, Jiankang Lu, Zhiqiang Zhou, Weihao Wang, Bingyao Bai, Fen Li, Chunlan Zhang

**Affiliations:** 1 College of Food Science and Engineering, Tarim University, Alar 843300, China; 15076833889@163.com (Y.W.); clw15937457327@163.com (L.C.); 18291852975@163.com (X.C.); 12010034@taru.edu.cn (J.L.); zhou2011519@sina.com (Z.Z.); zlzxwwh@126.com (W.W.); 120180028@taru.edu.cn (B.B.); fenli@taru.edu.cn (F.L.); 2 Production & Construction Group Key Laboratory of Special Agricultural Products Further Processing in Southern Xinjiang, Alar 843300, China; 3 Instrumental Analysis Center, Tarim University, Alar 843300, China; 4 Research Center of Food Colloids and Delivery of Functionality, College of Food Science and Nutritional Engineering, China Agricultural University, Beijing 100083, China

**Keywords:** chickpea starch, porous starch, enzymatic hydrolysis time, curcumin, in vitro digestion

## Abstract

To address the poor water solubility and low stability of curcumin, this study prepared chickpea porous starches at different enzymatic hydrolysis times (0, 2, 4, 6, and 8 h) through the synergistic action of α-amylase and glucoamylase and evaluated their performance as a natural carrier for curcumin. The results showed that as the enzymatic hydrolysis time lengthened, the pore size increased, dense micropores formed on the starch granule surface, and the specific surface area and pore volume increased significantly. Furthermore, the solubility, water-holding and oil-holding capacities, swelling power, and transparency of the porous starch were markedly improved, while the original C-type crystalline structure was maintained. Loading experiments revealed that the porous starch hydrolyzed for 8 h exhibited the strongest adsorption performance, achieving a curcumin encapsulation efficiency of up to 80.65%. These complexes not only significantly enhanced the thermal stability of curcumin but also preserved its excellent antioxidant activity (ABTS and DPPH radical scavenging capacities), while the carrier itself demonstrated enhanced resistance to in vitro digestion. This study provides a theoretical basis and practical reference for the development of novel legume-starch-based green delivery systems and for the efficient encapsulation of hydrophobic bioactive substances.

## 1. Introduction

Starch, the most abundant storage polysaccharide in plants, is organized into semi-crystalline granules in which amylose and amylopectin are arranged into alternating crystalline and amorphous lamellae, and this hierarchical architecture largely determines the functional behavior of starch in food systems [1]. Native starches isolated from different botanical sources differ markedly in granule morphology, crystalline type, amylose content, and physicochemical properties [2]. Chickpea (*Cicer arietinum* L.) is one of the most important food legumes worldwide, and starch is its principal component, accounting for approximately 37–50% of the dry seed weight. Compared with cereal starches, chickpea starch exhibits a characteristic C-type crystalline pattern, a relatively high amylose content (approximately 30–40%), restricted swelling, and high gelatinization temperatures, as well as a slow digestion rate that is desirable for low-glycemic foods [3]. These distinctive structural features make chickpea starch a promising substrate for the development of value-added modified starches.

Nevertheless, the compact granular structure of native chickpea starch results in low cold-water solubility and limited adsorption capacity, which restricts its industrial applications. Porous starch, a modified granular starch bearing abundant pores and channels while retaining granular integrity, can be produced by the partial hydrolysis of native granules with amylolytic enzymes below the gelatinization temperature [4]. Because enzymatic erosion proceeds preferentially through the less-ordered regions of the granule, the granular morphology is essentially preserved while the specific surface area and pore volume increase substantially, thereby improving the solubility, swelling power, and adsorption performance of the starch [5,6]. The mode of pore formation strongly depends on the catalytic pattern of the enzyme; most researchers adopt glucoamylase and α-amylase preparation of porous starches from corn, potato, etc., through synergistic action [4,7,8].

Among the structural attributes of porous starch, pore size is of particular importance because it governs the diffusion and accessibility of guest molecules and thus largely determines the adsorption and encapsulation performance of the granules [6,9]. Owing to these features, porous starch has been widely explored as a natural, food-grade carrier for essential oils [10], probiotics [11], and other bioactive compounds, and nitrogen physisorption analysis has become a standard tool for quantifying its specific surface area and pore size distribution [12]. However, previous studies have focused mainly on cereal and tuber starches such as corn, rice, potato, and cassava [5,7,8]; systematic information on chickpea porous starch remains scarce, and, in particular, the relationship among the enzymatic hydrolysis time, pore structure, and physicochemical properties of chickpea porous starch has not been fully elucidated.

Therefore, in this study, chickpea porous starches with different pore sizes were prepared by controlling the hydrolysis time (0, 2, 4, 6, and 8 h) during the synergistic action of α-amylase and glucoamylase. The pore structure, crystalline and short-range molecular order, and pasting behavior of the starches were characterized by scanning electron microscopy (SEM), nitrogen adsorption–desorption analysis (BET), X-ray diffraction (XRD), Fourier transform infrared spectroscopy (FTIR), and rapid viscosity analysis (RVA), and their physicochemical properties, including solubility, swelling power, water- and oil-holding capacities, and transparency, were systematically evaluated. Furthermore, the potential of the resulting porous starches as natural carriers was assessed using curcumin as a model hydrophobic bioactive compound. This work is expected to clarify how the enzymatic hydrolysis time regulates the pore structure and, consequently, the physicochemical properties of chickpea porous starch and to provide a theoretical basis for the rational design of legume-starch-based porous materials and delivery systems.

## 2. Materials and Methods

Chickpeas were purchased from the Alar market in China. Curcumin was purchased from Shanghai Maclean’s Biochemical Technology Co., Ltd. (Shanghai, China). Glucoamylase (100,000 U/g) and α-amylase (aspergillus oryzae) (100,000 U/g), simulated saliva fluid (SSF, catalog number R30093), simulated gastric fluid (SGF, catalog number R27248) and simulated intestinal fluid (SIF, catalog number R30384) were obtained from Yuanye Bio-Technology Co., Ltd. (Shanghai, China). Alkaline protease (200,000 U/g) was purchased from Beijing Solarbio Science & Technology Co., Ltd. (Beijing China). All other chemicals were of analytical grade.

### 2.1. Extraction of Chickpea Starch

Chickpeas were washed, dried at room temperature, ground into flour, and passed through a 100-mesh sieve. The chickpea flour was mixed with 0.1% NaOH solution at a solid-to-liquid ratio of 1:5 (g/mL), the pH was adjusted to 10.84, and 0.1% alkaline protease (200,000 U/g) was added. The slurry was incubated in a constant-temperature water bath at 42 °C for 90 min. After hydrolysis, the slurry was centrifuged at 4000 r/min for 10 min at 25 °C; the supernatant was discarded, and the precipitate was washed with deionized water and centrifuged again under the same conditions, repeated three times. The precipitate was dried in an oven at 40 °C to obtain the chickpea starch extract. Upon determination of starch purity by acid–base titration, the starch content reaches 90% [13].

### 2.2. Preparation of Chickpea Porous Starch

Chickpea starch (2 g) was weighed, mixed with 18 mL of phosphate-buffered solution (pH 5.5), and preheated in a water bath at 55 °C for 15 min. A 5% composite enzyme (α-amylase, 100,000 U/g:glucoamylase, 100,000 U/g = 1:6 U:U) was then added, total enzyme activity 1000 U/g, and the mixture was reacted in a magnetically stirred water bath at 55 °C for different reaction times (0, 2, 4, 6, and 8 h); 0.1 mM NaOH inactivated the enzyme and adjusted the pH to 7. After the reaction, the mixture was centrifuged at 8000 r/min for 10 min; the supernatant was discarded, and the precipitate was washed with deionized water and centrifuged again under the same conditions, repeated three times. The precipitate was dried in an oven at 40 °C to obtain chickpea porous starches, designated PS-0, PS-2, PS-4, PS-6, and PS-8, respectively.

### 2.3. Preparation of Chickpea Porous Starch–Curcumin Complexes

Curcumin was dissolved in anhydrous ethanol and centrifuged at 3000 r/min for 10 min to remove undissolved crystals. The starches (PS-0, PS-2, PS-4, PS-6, and PS-8) were separately suspended in distilled water at 10 mg/mL and sonicated in an ultrasonic cleaner to obtain stable dispersions. Under magnetic stirring (150 rpm, 30 °C), the curcumin solution was slowly added to each starch suspension (1%) (curcumin:starch = 1:1, *v*:*v*) to prepare curcumin–porous starch composite samples. The resulting suspensions were ultrasonicated in an ice bath at a rated output power of 300 W for 5 min to enhance curcumin adsorption. Then they were centrifuged at 3000 r/min for 10 min, the supernatant was discarded to remove free curcumin, and it was washed with distilled water and centrifuged three times to prevent the encapsulation efficiency from expanding. The samples were then freeze-dried to obtain starch–curcumin microspheres (PS-0-C, PS-2-C, PS-4-C, PS-6-C, and PS-8-C) and stored at −20 °C until use.

### 2.4. Structural Characterization of Chickpea Porous Starch

#### 2.4.1. Scanning Electron Microscopy (SEM)

Samples were uniformly dispersed onto conductive adhesive tape, sputter-coated with gold, and observed under a scanning electron microscope at appropriate magnifications to capture representative granule morphologies.

#### 2.4.2. N_2_ Adsorption–Desorption Test (BET)

Nitrogen adsorption–desorption measurements were performed on a specific surface area and porosity analyzer at 77 K. High-purity nitrogen (>99.99%) was used to establish the adsorption–desorption isotherms. Each starch sample was dehydrated at 100 °C for 12 h and degassed at 100 °C for 10 h, and the sample tube was placed at liquid nitrogen temperature (−196 °C) for testing. The specific surface area was calculated using the BET equation over a relative pressure range (P/P_0_) of 0.05–0.30.

#### 2.4.3. Fourier Transform Infrared Spectroscopy (FTIR)

A 1.0 mg portion of the dried sample was mixed with 150 mg of potassium bromide, ground into a uniform powder, and pressed into a thin pellet. Spectra were collected using an FT-IR spectrometer over the range of 4000–400 cm^−1^ at a resolution of 4 cm^−1^ with 32 scans. The absorbance ratio at 1047/1022 cm^−1^ (R1047/1022) was calculated from the deconvoluted spectra in the 1200–900 cm^−1^ region as an index of the short-range molecular order of starch, following the methods of van Soest et al. [14] and Sevenou et al. [15].

#### 2.4.4. X-Ray Diffraction Patterns (XRD)

Samples were analyzed by X-ray diffraction under the following conditions: copper target, voltage 40 kV, current 40 mA, and CuKα radiation; scanning range 5–45°, step size 0.02°, and scanning speed 4°/min. The relative crystallinity (%) of the samples was calculated as the ratio of the crystalline peak area to the total area of the diffractogram after peak fitting using MDI Jade 6 software, according to the methods described by Nara and Komiya [16,17] and Cheetham and Tao [16].

### 2.5. Determination of Properties of Chickpea Porous Starch

#### 2.5.1. Determination of Solubility and Swelling Power

An appropriate amount of starch was weighed and prepared as a 2% starch suspension. The suspension was stirred in a constant-temperature magnetic water bath at 55, 65, 75, 85, or 95 °C for 30 min. After cooling to room temperature, the suspension was transferred to a centrifuge tube of mass m_1_ and centrifuged at 4000 r/min for 10 min. The supernatant was poured into a Petri dish of mass m_2_ and dried in an oven at 105 °C to constant weight (recorded as m_3_), and the centrifuge tube with the precipitate was weighed (recorded as m_4_). Calculations were performed according to Formulas (1) and (2).

(1) Solubility: S % = (m_3_ − m_2_)/w × 100%

where m_2_ is the mass of the Petri dish (g); m_3_ is the mass of the Petri dish after drying to constant weight (g); and w is the mass of the starch sample (g).

(2) Swelling Power: B % = (m_4_ − m_1_)/[w × (1 − S)] × 100%

where m_4_ is the mass of the centrifuge tube with the precipitate (g); m_1_ is the mass of the centrifuge tube (g); w is the mass of the starch sample (g); and S is the solubility (substitute with fractions).

#### 2.5.2. Determination of Starch Pasting Curves (RVA)

The pasting properties of the starch samples were measured using a RVA 4500 Rapid Visco Analyzer, Perten Instruments Pty Ltd., Macquarie Park, NSW, Australia. A 1.0 g starch sample was mixed with 25 mL of water, initial thermal equilibrium at 50 °C was held for 1 min, and then the temperature was increased dynamically at a rate of 12 °C per minute while stirring at a speed of 160 revolutions per minute until the temperature rose to 95 °C. Using Thermocline Version 3.15 (Perten Instruments Australia, Macquarie Park, NSW, Australia). After that, the sample was kept at 95 °C for 2.5 min; finally, it was cooled down to 50 °C at a rate of 12 °C per minute, and kept at 50 °C for 2 min. Peak viscosity (cP), trough viscosity (cP), final viscosity (cP), breakdown value (cP), setback value (cP), and pasting temperature (°C) were recorded.

#### 2.5.3. Determination of Freeze–Thaw Stability

Starch was weighed and prepared as a 6% starch suspension, gelatinized at 95 °C for 20 min, then removed, shaken, and cooled to room temperature. The sample was stored in a refrigerator at −18 °C for 24 h and then thawed naturally at room temperature for 6 h. After centrifugation at 4000 r/min for 15 min, the supernatant was removed. The water separation rate was calculated according to Formula (3).

(3) Water separation rate (%) = (M − W)/M × 100%

where M is the mass of the starch paste (g) and W is the mass of the precipitate (g).

#### 2.5.4. Determination of Transparency

A 1% starch suspension was prepared, heated in a 95 °C water bath for 20 min, and cooled to room temperature. Using distilled water as the blank, the transmittance of the sample was measured at 620 nm with a spectrophotometer.

#### 2.5.5. Determination of Water-Holding Capacity and Oil-Holding Capacity

Approximately 0.2 g of starch was weighed, and 5 mL of deionized water or soybean oil was added. The mixture was vortex-mixed, incubated in a 40 °C water bath for 30 min, and then centrifuged at 4000 r/min for 10 min. The unabsorbed water or oil in the upper layer was decanted, and the water/oil-holding capacity was calculated according to Formula (4).

(4) Water/Oil Holding Capacity (g/g) = (m_2_ − m_1_ − m_0_)/m_0_

where m_0_ is the mass of the sample (g); m_1_ is the mass of the centrifuge tube (g); and m_2_ is the total mass of the centrifuge tube, sample, and adsorbed water/oil (g).

#### 2.5.6. In Vitro-Simulated Digestion

A 2 g sample was mixed with 5 mL of simulated saliva and incubated in a shaking water bath at 37 °C for 2 min. The saliva-digested sample was then combined with simulated gastric fluid at a 1:1 (*v*/*v*) ratio and incubated at pH 3.0 and 37 °C for 2 h to simulate gastric digestion. The resulting mixture was collected as the salivary–gastric digest. Simulated intestinal fluid was then added at a 1:1 (*v*/*v*) ratio, the pH was adjusted to 8, and the mixture was incubated at 37 °C for 2 h to simulate intestinal digestion. At 15, 30, 60, and 120 min during each digestion stage, 1 mL of sample was withdrawn with a pipette. and the enzymes were inactivated by heating at 95 °C for 5 min.

When measuring starch fractions (RDS, SDS, RS), the sampling time is set at 20, 120 and 240 min throughout the digestion stage.

The measurement method of starch digestibility adopts the DNS reagent method. The absorbance is measured at a wavelength of 540 nm with an ultraviolet spectrophotometer, and the measured value is substituted into the standard curve y = 16.241x − 0.0533, R^2^ ≥ 0.99. Then the digestibility is calculated using Formula (5), and the starch fractions (RDS, SDS, RS) are calculated using Formulas (6)–(8).

(5) *Digestibility*(%) = *x* × *V* × *N* × 0.9/(*m* × 1000) × 100%

where x is the sugar content (mg/mL), V is the total volume before each sampling (mL), *N* is the dilution factor, 0.9 is the conversion coefficient between glucose and starch (starch molecular weight/glucose molecular weight = 162/180), and m is the total mass of the starch sample (g).

(6) $$\mathrm{RDS}=G_{20\min}\times \frac{0.9}{m}\times 100\%$$

(7) SDS = [(*G*_120_*_min_* × 0.9) − (*G*_20_*_min_* × 0.9)]/*m* × 100%

(8) $$\mathrm{RS}=\frac{\left[m-G_{240\min}\times 0.9\right]}{m}\times 100\%$$

where Gx is the sugar content (mg/mL) at the sampled time, m is the total mass of the starch sample (g), and 0.9 is the conversion coefficient between glucose and starch (starch molecular weight/glucose molecular weight = 162/180).

The measurement method for curcumin release is as follows: Add 1 mL of alcohol to the taken sample to inactivate the enzyme, then measure the absorbance at a wavelength of 426 nm using an ultraviolet spectrophotometer, and substitute the value into the standard curve y = 0.0312x − 0.0205, R^2^ ≥ 0.99. Then the release rate is calculated using Formula (9).

(9) Release rate(%) = *x* × *V* × *N*/(*m* × 1000) × 100%

where x is the curcumin content (mg/mL), V is the total volume before each sampling (mL), *N* is the dilution factor, and m is the mass of curcumin in the complex (g).

### 2.6. Characterization of Porous Starch–Curcumin (PS-C) Complexes

#### 2.6.1. Encapsulation Efficiency

A 1% starch suspension was prepared. The ethanolic curcumin solution was mixed with the starch suspension at a 1:1 ratio and ultrasonicated for 5 min. Free curcumin was separated by centrifugation (3000 r/min, 10 min), the absorbance of the supernatant at 426 nm was measured using a spectrophotometer, and the curcumin content was calculated according to the standard curve y = 0.0312x − 0.0205, R^2^ ≥ 0.99. The encapsulation efficiency (EE %) of curcumin in the starch carrier was calculated according to Formula (10).

(10) EE(%) = (M_t_ − M_m_)/M_t_ × 100%

where M_t_ is the total mass of curcumin added and M_m_ is the mass of unadsorbed curcumin.

#### 2.6.2. Stability of Curcumin

The 1% starch suspensions were mixed with the ethanolic curcumin solution at a 1:1 ratio and incubated in a water bath under light-proof conditions at 25 °C or 60 °C. After 90 min, samples were collected to determine the curcumin content.

#### 2.6.3. Antioxidant Activity

A 100 μL aliquot of DPPH· ethanolic solution (50.0 μg/mL) was mixed with 100 μL of PS-C sample solution (0.5 mg/mL) and incubated at 25 °C in the dark for 30 min. The absorbance of the mixture was then measured at 517 nm.

(11) SA_DPPH_(%) = (A_0_ − A_C_)/A_0_ × 100%

where A_0_ is the absorbance of the control (methanol in place of the sample) and A_C_ is the absorbance of the sample.

ABTS (200 mg), potassium persulfate (34.4 mg), and deionized water (50 mL) were thoroughly mixed and reacted in the dark at room temperature for 24 h to obtain the ABTS stock solution. A working solution was prepared by diluting the stock solution with ethanol until the absorbance reached 0.70 ± 0.02. Then, 3.6 mL of the ABTS working solution was combined with 0.4 mL of sample solution (0.5 mg/mL) and allowed to react in the dark at room temperature for 30 min, and the absorbance was measured at 734 nm.

(12) SA_ABTS_(%) = (A_0_ − A_C_)/A_0_ × 100%

where A_0_ is the absorbance of the control (methanol in place of the sample) and A_C_ is the absorbance of the sample.

#### 2.6.4. Thermal Stability

Porous starch and starch–curcumin microspheres were heated from 0 to 600 °C at a rate of 10 °C/min using a thermogravimetric analyzer (TGA4000, PE).

### 2.7. Statistical Analysis

Statistical analyses were performed using one-way analysis of variance (ANOVA) with IBM SPSS Statistics 25 (SPSS Inc., Chicago, IL, USA). software. For experiments involving a single factor, data were analyzed by one-way analysis of variance (ANOVA), and involving two factors, data were analyzed by two-way ANOVA. Mean comparisons were performed using Duncan’s multiple range test at *p* ≤ 0.05. All measurements were performed in triplicate using independently prepared samples, and the results are expressed as mean ± standard deviation (SD). All theillustration drawings were constructed using OriginPro 2025 (OriginLab Co., Northampton, MA, USA).

## 3. Results

### 3.1. Structural Analysis of Chickpea Porous Starch

#### 3.1.1. Scanning Electron Microscopy Analysis (SEM)

**Figure 1:**
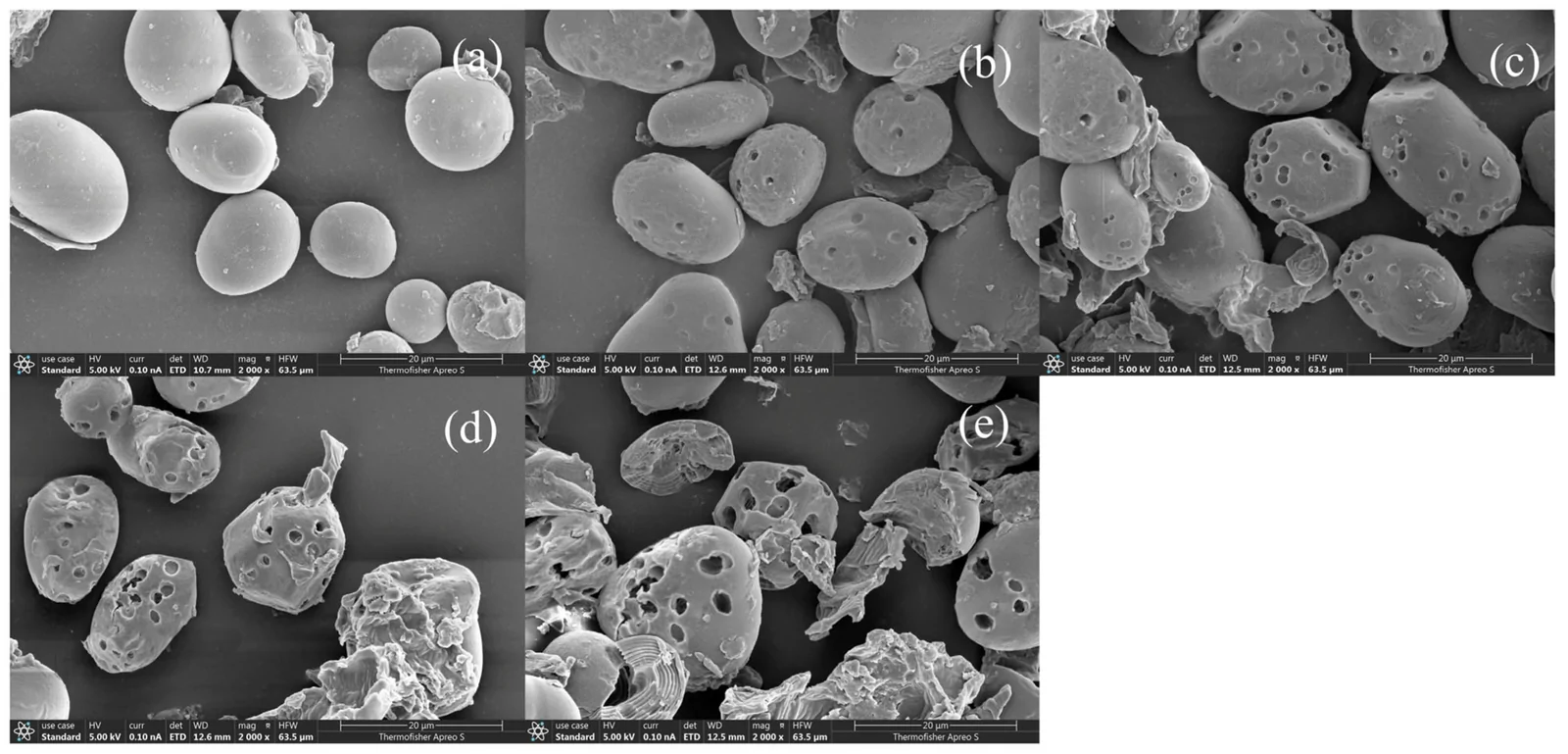
Scanning electron micrographs of chickpea porous starch. (**a**) PS-0; (**b**) PS-2; (**c**) PS-4; (**d**) PS-6; (**e**) PS-8.

SEM images of the porous starches prepared at different enzymatic hydrolysis times are shown in Figure 1. Native chickpea starch granules are mostly elliptical, with well-defined edges, smooth surfaces, and intact structures [18]. After 4 h of enzymatic hydrolysis, small pores begin to form on the starch surface (Figure 1c). As the hydrolysis time increases, the pores become denser and extend into the granule interior, resulting in a rougher surface. The SEM image of PS-8 (Figure 1e) shows partial collapse of the granule surface, which may be attributed to the hydrolysis of amylopectin in the relatively loose amorphous regions that are preferentially degraded by the enzymes. This gradual enzymatic process ultimately leads to the formation of a porous structure inside the starch granules [19]. Furthermore, enzyme treatment increases the relative amylose content, promoting a denser crystalline structure. The enhanced crystallinity increases the resistance of the granules to enzymatic degradation, thereby increasing the resistant starch content [20]. Therefore, enzymatic modification not only improves the adsorption performance of starch but also enhances its potential as a resistant starch, making it highly valuable for applications in fields such as food and medicine.

#### 3.1.2. Specific Surface Area and Pore Size Distribution Analysis

Table 1 presents the nitrogen adsorption parameters, including specific surface area, pore volume, and average pore size, of native chickpea starch and the porous starches prepared with different reaction times. Owing to the absence of pore structures, native chickpea starch has a relatively small specific surface area of 0.156 m^2^/g. Compared with native starch, the specific surface areas of all porous starch samples increased. Among them, the porous starch hydrolyzed for 8 h exhibited the largest specific surface area (0.367 m^2^/g), as well as the largest pore volume and average pore size. These results indicate that enzymatic hydrolysis proceeds rapidly at the early stage, and that after 8 h of hydrolysis the starch granules undergo more extensive hydrolysis while largely retaining their integrity, resulting in strong adsorption performance, which is consistent with the SEM observations.

**Figure 2:**
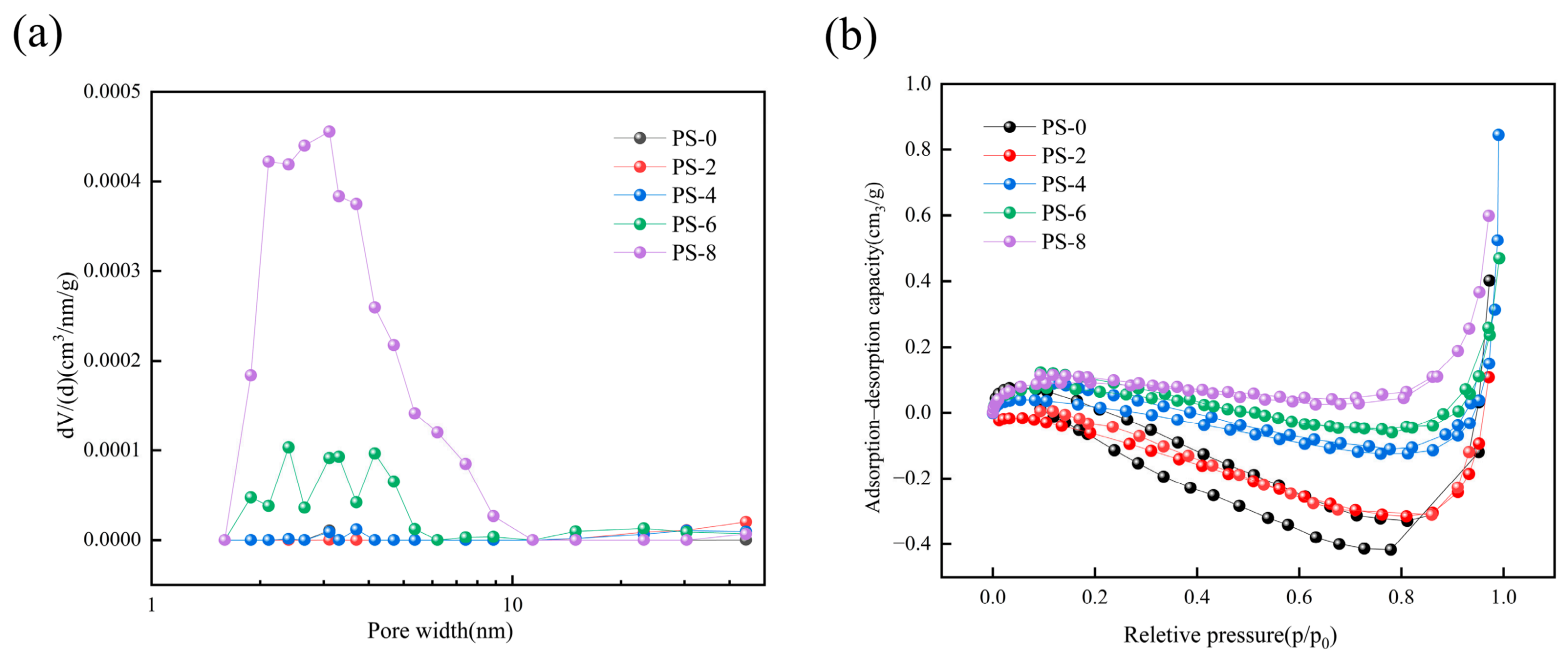
The differential curves of pore volume distribution (**a**) and the adsorption–desorption curves (**b**) of porous starches prepared at different enzymatic hydrolysis times.

Figure 2a depicts the differential pore volume distribution curves. The pores were classified into three types: micropores (diameters < 2 nm), mesopores (2 ≤ diameters ≤ 50 nm), and large pores (diameters greater than 50 nm) [21]. Wider ranges of peaks in the differential curves of pore volume distribution indicate more pores of different sizes in the pore width range, and smaller ranges of peaks indicate more uniform pore size of the starch. The intensity of the peak indicates the number of pores. The greater the pore volume distribution, the more pores at that pore size there are [22]. Distinct peaks can be observed in PS-8 and PS-6. The peaks of other porous starch samples are very weak. It can be seen that the extension of enzymatic hydrolysis time promotes the increase in pore sizes of porous starch. It can also be seen that the peak of the PS-8 curve covers a wider range, which indicates more pores and a more uniform pore size distribution. The adsorption–desorption curves of porous starches are shown in Figure 2b. The porous starch adsorption isotherms are type IV. Type IV is the hysteresis phenomenon due to the appearance of capillary aggregates. The isotherm of desorption is not consistent with the isotherm of adsorption, and it is higher than the isotherm of adsorption, resulting in adsorption hysteresis and a hysteresis loop, which is characteristic of mesoporous materials [23].

#### 3.1.3. Fourier Transform Infrared Spectroscopy Analysis of Porous Starch (FTIR)

**Figure 3:**
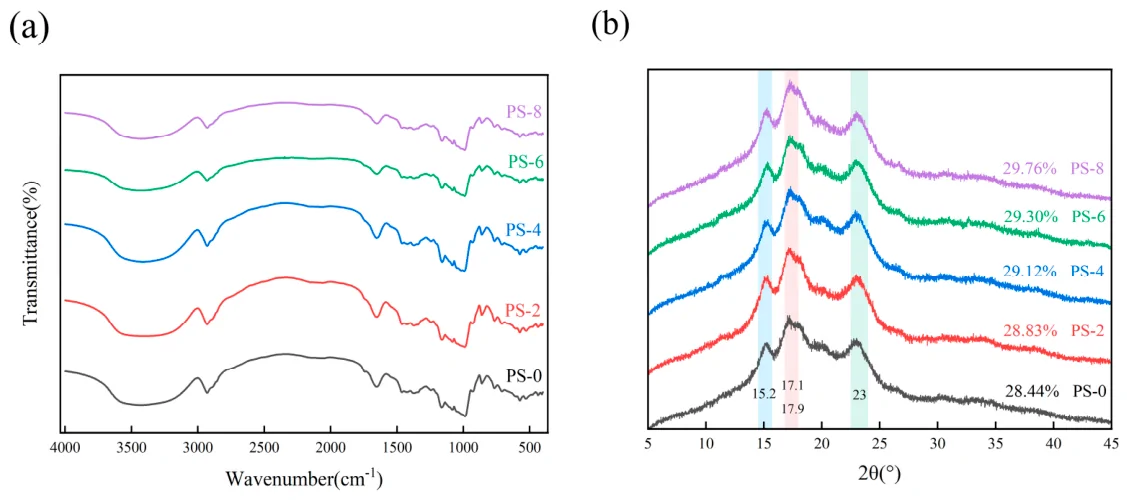
FTIR (**a**) and X−ray diffraction patterns (**b**) of porous starches prepared at different enzymatic hydrolysis times.

As shown in Figure 3a, the characteristic absorption peaks of native chickpea starch and the porous starches prepared with different reaction times are essentially identical in position. Across all starch samples, the peaks at 1148, 1072, and 1017 cm^−1^ are assigned to the coupled C–O and C–O–C stretching vibrations of the glycosidic linkages and the anhydroglucose ring [16]; the peak at 2900 cm^−1^ is attributed to C–H stretching; and the peak near 3400 cm^−1^ is attributed to the stretching of –OH groups within the starch granules.

α-Amylase and glucoamylase act synergistically on the granule surface and amorphous regions of starch, preferentially hydrolyzing α-1,4-glycosidic bonds. The FTIR spectra show clear trends in the 1000–1150 cm^−1^ region: the absorbance at 1047 cm^−1^ characterizes the crystalline region, whereas that at 1022 cm^−1^ reflects the amorphous structure [24,25]. The intensity of the C–O–C glycosidic bond absorption peak continuously weakens, indicating increased cleavage of the starch main chains. Meanwhile, the O–H stretching peak in the 3200–3600 cm^−1^ region gradually broadens, reflecting an increase in the number of hydroxyl groups participating in hydrogen bonding and a transition of the hydrogen bond network from ordered to disordered [26]. These changes correspond directly to the enhanced surface hydrophilicity of the starch granules and the improved wettability of the micropore surfaces.

#### 3.1.4. X-Ray Diffraction Analysis (XRD)

Starch is a high-molecular-weight polymer whose crystalline and amorphous regions exhibit characteristic X-ray diffraction features: the crystalline regions produce sharp peaks, whereas the amorphous regions produce diffuse scattering. The crystal type of starch granules can be identified from the positions of the diffraction peaks. The X-ray diffraction patterns of the different porous starch samples are shown in Figure 3b. Native chickpea starch exhibits diffraction peaks near 15°, 17°, and 23°, corresponding to a C-type crystalline structure [27,28]. Compared with native starch, the enzymatically hydrolyzed porous starch samples show identical peak positions and a similar, C-type crystal structure, indicating that the crystalline form remained unchanged after enzymatic treatment [29]. The relative crystallinity increased from 28.44% to 29.76%, which is attributed to enzymatic hydrolysis acting on the amorphous regions [30]: hydrolases such as α-amylase and glucoamylase more readily access and cleave the non-crystalline regions, leading to their hydrolysis and a consequent increase in relative crystallinity [8].

### 3.2. Characteristic Analysis of Porous Starch from Chickpeas

#### 3.2.1. Solubility and Swelling Power Analysis

**Figure 4:**
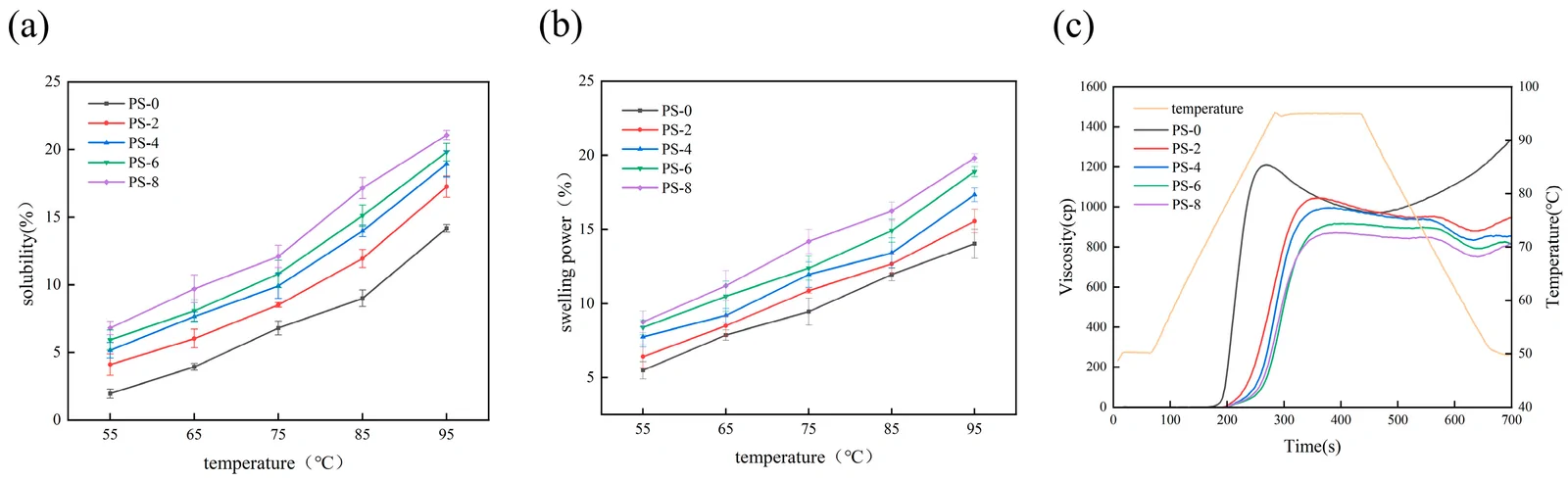
Solubility (**a**) and swelling power (**b**) of chickpea porous starches at different temperatures; pasting viscosity curves of porous starch (**c**). Values reported are mean of triplicate determinations; error bars are standard deviations.

As shown in Figure 4a,b, the solubility and swelling power of the porous starches prepared at different enzymatic hydrolysis times increased with temperature. And as the enzymatic hydrolysis time extended, the pore size increased, and solubility and swelling degree gradually increased. This is mainly attributed to the leaching of amylose from the non-crystalline regions of the starch after enzymatic hydrolysis, which increases solubility [4,7]. In addition, the porous structures formed on the granule surface increased the specific surface area and pore volume, thereby endowing the porous starch with higher swelling power [5].

#### 3.2.2. Changes in Starch Pasting Viscosity Properties

The pasting properties of the different porous starch samples were measured using a Rapid Visco Analyzer. As shown in Table 2, the peak viscosity, trough viscosity, final viscosity, breakdown value, and setback value of the porous starches were all lower than those of native chickpea starch, whereas the pasting temperature was higher. This may be because enzymatic treatment shortens the starch molecular chains and increases solubility, leading to decreases in peak, trough, and final viscosities. A larger breakdown value indicates poorer thermal stability, whereas a smaller value indicates stronger thermal stability. The setback value (SB) is related to the recrystallization and rearrangement of starch during cooling [31]; a lower SB value indicates slower formation of helical structures and gels and thus better anti-retrogradation performance [32]. The increase in starch suspension viscosity arises from the swelling of starch granules during heating, as swollen granules form a network through close contact. Enzymatic hydrolysis promotes granule disintegration and the leaching of soluble molecules, thereby reducing viscosity.

As shown in Figure 4c, the PV and TV of the starch decreased from 1131.25 ± 89.73 mPa·s and 901.75 ± 70.34 mPa·s to 842.50 ± 82.29 mPa·s and 715.50 ± 59.26 mPa·s, respectively. The viscosity of the porous starch was lower than that of the native starch and decreased gradually as the enzymatic hydrolysis time prolonged and the pore size increased, which is attributed to the disruption of the granular framework and the loss of soluble molecules, weakening the swelling and thickening capacity. These results are consistent with the XRD analysis.

#### 3.2.3. Freeze–Thaw Stability Analysis

**Figure 5:**
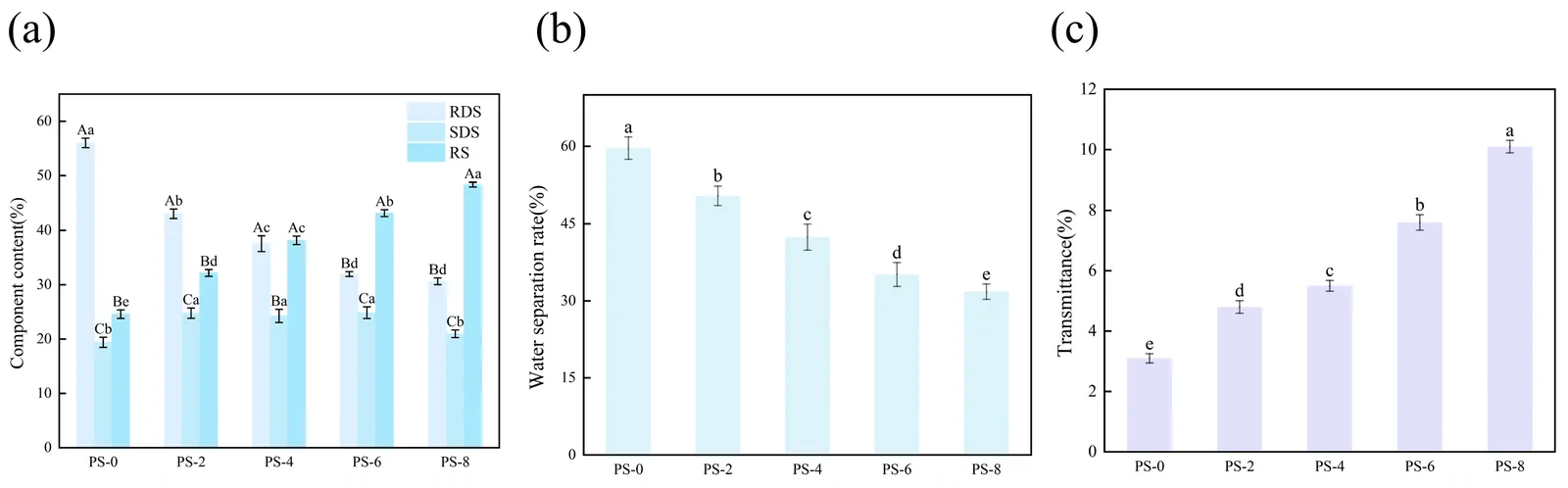
Starch component content (**a**), water separation rate (**b**) and transparency (**c**) of porous starches prepared at different enzymatic hydrolysis times. Different letters (a–e and A–C) indicate significant differences (*p* < 0.05) between samples; error bars are standard deviations.

The water separation rate (syneresis) refers to the percentage of free water released from a gel or gelatinized system during centrifugation, standing, or storage, and serves as an indicator of the physical stability and water-holding network integrity of the system. As shown in Figure 5b, the water separation rate of the porous starch enzymatic hydrolyzed for 8 h (PS-8) decreased to 31.78%, a reduction of 27.87% compared with native starch [33]. Native chickpea starch has a dense structure and a smooth surface, and its interior is composed of closely packed crystalline and amorphous regions formed by amylose helices and amylopectin clusters [34]. During cooling and storage, amylose tends to retrograde, forming rigid double-helix crystalline regions that expel large amounts of bound water and exacerbate syneresis [35]. In contrast, enzymatic hydrolysis introduces pores on the granule surface of porous starch. These pores not only significantly increase the number of molecular contact sites but also fully expose the hydrophilic hydroxyl groups (–OH) on the granule surface, markedly enhancing the hydrogen bonding capacity [36]. More importantly, the pore structure changes the state of water: capillary action draws water into the pores, reducing its mobility and stabilizing the hydrogen bond network, so that the water is difficult to separate under gravity or centrifugal force [37].

#### 3.2.4. Transparency Analysis

Transparency is usually quantified by light transmittance, the proportion of light that passes directly through a gelatinized starch gel or film without being scattered or absorbed. As shown in Figure 5c, the transparency of native starch is only 3%, whereas that of the porous starch after 8 h of reaction increases to 9.4% [38]. This is mainly because the dense granules of native starch undergo incomplete swelling and heterogeneous fragmentation during heating and gelatinization, forming large numbers of irregular fragments and amylopectin clusters of 100–500 nm that act as strong scattering centers and reduce light transmittance [39]. In contrast, the α-1,4-glycosidic bonds of the enzymatically hydrolyzed porous starch are cleaved, releasing the originally entrapped amylose segments and promoting their more uniform dispersion in the continuous phase during gelatinization, which reduces light scattering and markedly increases transparency [40].

#### 3.2.5. Water- and Oil-Holding Capacity Analysis

**Figure 6:**
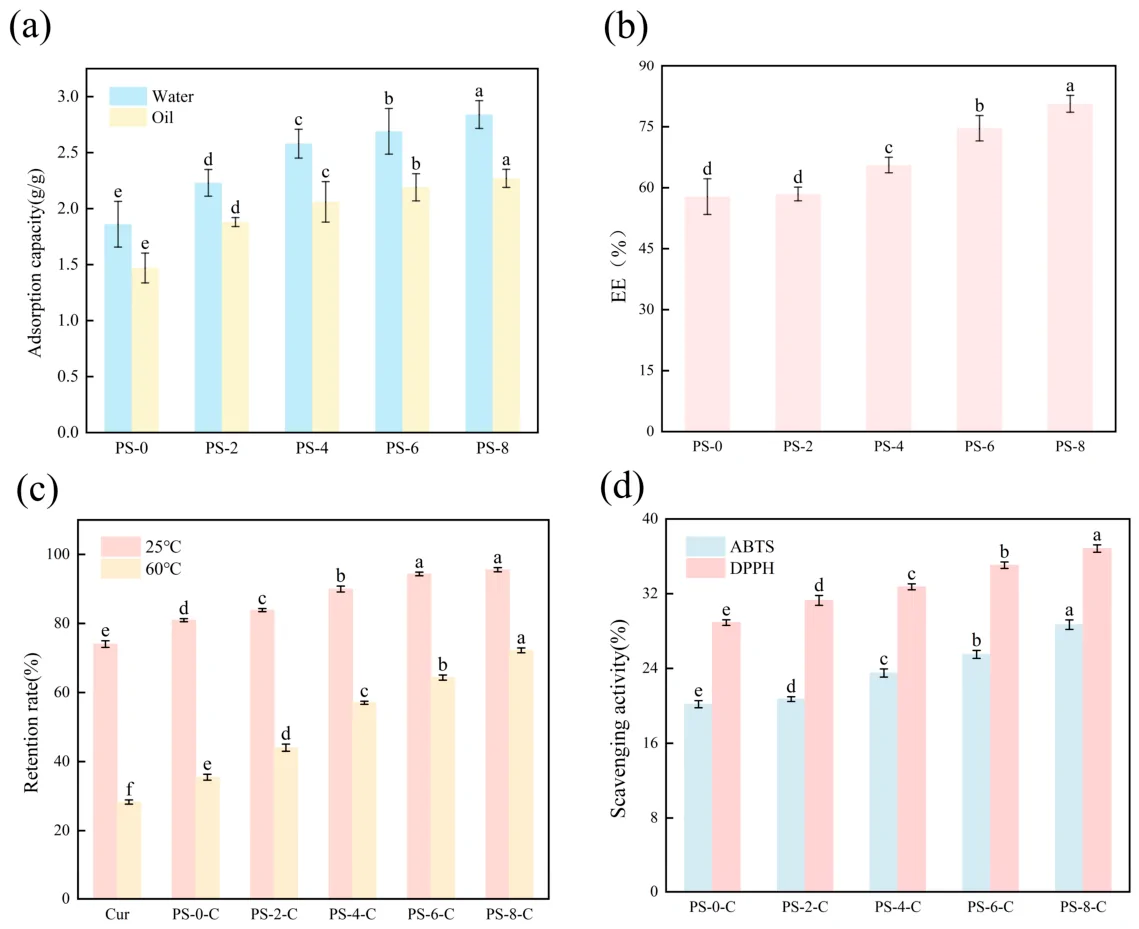
Water- and oil-holding capacity of porous starches prepared at different enzymatic hydrolysis times (**a**); encapsulation efficiency of curcumin by porous starch (**b**); curcumin retention rates of the porous starch−curcumin complexes at 25 °C and 60 °C (**c**); free radical scavenging activities of the porous starch−curcumin microspheres (**d**). Different letters (a–f) indicate significant differences (*p* < 0.05) between samples; error bars are standard deviations.

As shown in Figure 6a, both the water- and oil-holding capacities of the porous starch increased. Because its dense structure restricts the penetration of water molecules, native starch has a water-holding capacity of only 1.8 g/g, whereas after 8 h of enzymatic treatment, the water-holding capacity of the porous starch enzymatic hydrolyzed for 8 h (PS-8) reached 2.6 g/g. This is because enzymatic hydrolysis creates micropores on the granule surface, allowing water molecules to enter more easily [41]. The water-holding capacity is negatively correlated with the water separation rate: a higher water-holding capacity means that more water is effectively integrated into the gel network, reducing the proportion of free water and significantly improving the stability of the system [29].

Native chickpea starch has a low oil-holding capacity because its hydrophilic surface is incompatible with non-polar oils. The pores formed on the granule surface after enzymatic treatment provide space for oil retention; in addition, some amylose chains expose hydrophobic helical cavities during enzymatic hydrolysis, further enhancing oil adsorption [42,43].

### 3.3. Analysis of the Results of Porous Starch-Curcumin (PS-C) Complexs

#### 3.3.1. Curcumin Encapsulation Efficiency Analysis

As shown in Figure 6b, the encapsulation efficiency of curcumin increases markedly for porous starches prepared with enzymatic hydrolysis times of 4 h or longer, reaching 80.65% for the porous starch hydrolyzed for 8 h (PS-8), which indicates that the porous starch possesses good encapsulation performance. Porous starch has a large specific surface area and high porosity and thus a high adsorption capacity [44], which is consistent with the BET results.

#### 3.3.2. Analysis of Curcumin Protection by Porous Starch

Curcumin is prone to degradation into vanillin, ferulic acid, and other products upon heat treatment [45], which is detrimental to its application in the food industry [46]. As shown in Figure 5c, at both room temperature (25 °C) and high temperature (60 °C), the curcumin retention rates of the complexes are significantly higher than that of free curcumin, and the retention rates of all samples at 60 °C are lower than those at 25 °C, indicating that the porous starch–curcumin complexes protect curcumin from thermal degradation [47,48]. This protection arises because curcumin is encapsulated within the pores of the porous starch and interacts with its hydroxyl groups via hydrogen bonding [49], which inhibits thermal degradation [50]. Consequently, the complexes exhibit much higher curcumin retention than free curcumin, which, lacking carrier protection, is susceptible to degradation by temperature and other environmental factors [51].

#### 3.3.3. Antioxidant Activity Analysis

Curcumin is a potent antioxidant capable of effectively scavenging DPPH and ABTS free radicals. Chickpea starch has almost no antioxidant capacity [52]. Maintaining its antioxidant capacity and ensuring its effective release are therefore crucial for porous starch–curcumin microspheres [53,54]. As shown in Figure 6d, the ABTS and DPPH radical scavenging abilities of PS-0-C are only 20.36% and 28.28%, respectively, which is attributed to the low curcumin loading of the non-porous starch, in which curcumin is retained only on the granule surface. The free radical scavenging abilities (ABTS and DPPH) of PS-2-C, PS-4-C, PS-6-C, and PS-8-C are significantly higher than those of PS-0-C, indicating that these porous starch–curcumin microspheres possess excellent antioxidant activity. The DPPH scavenging trends of the different microspheres are similar to their ABTS scavenging trends. Together with the encapsulation efficiency results, these findings indicate that the modified porous starch can not only adsorb curcumin but also preserve most of its antioxidant activity.

#### 3.3.4. Fourier Transform Infrared Spectroscopy Analysis of Porous Starch-Curcumin (PS-C) Complexs (FTIR)

**Figure 7:**
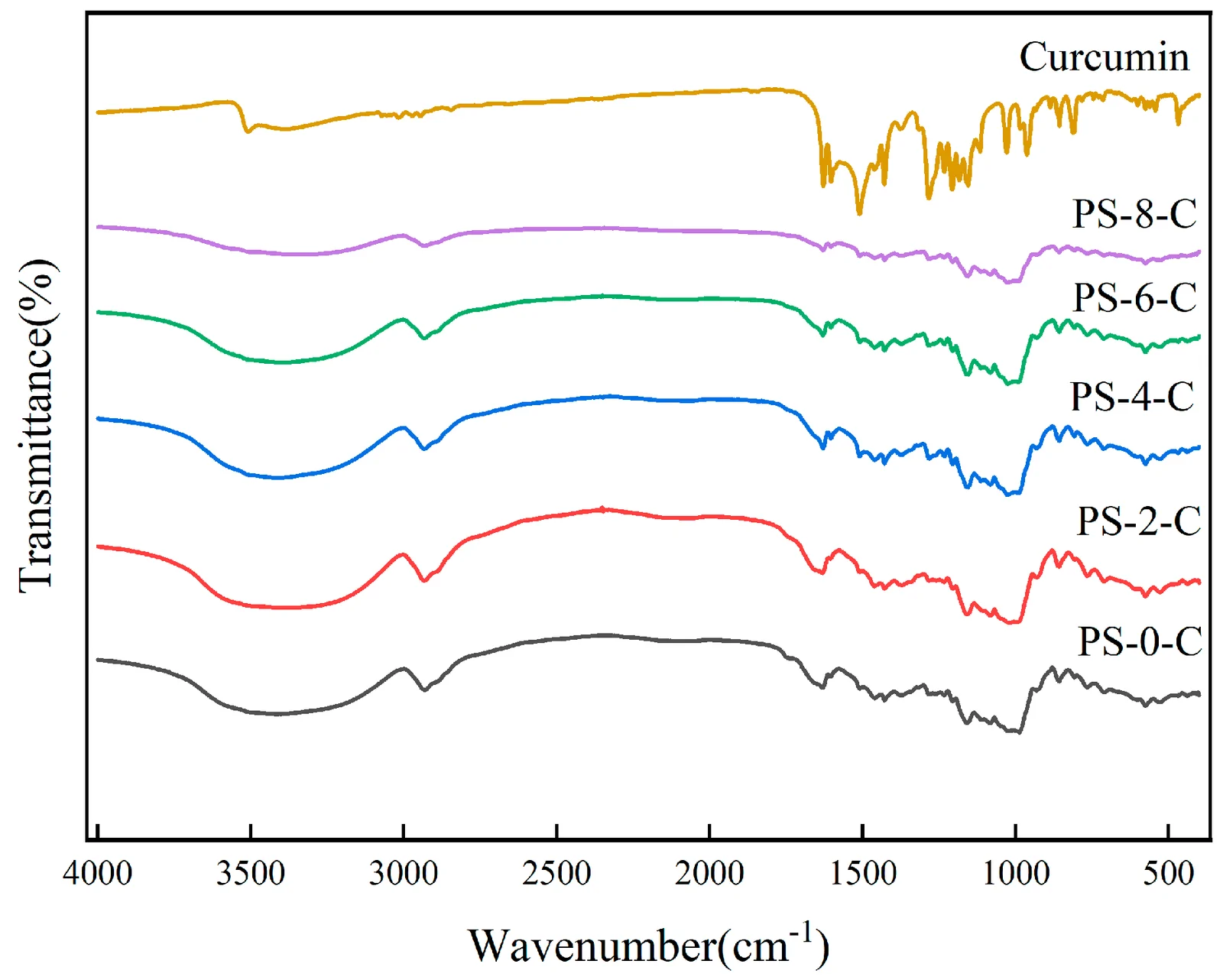
FTIR of the porous starch−curcumin microspheres.

As shown in Figure 7, in the curcumin spectrum, sharp absorption bands are observed at 3520, 1602, 1510, 1282, and 981 cm^−1^ (C-O-C stretching vibration), attributed to -OH, C=O, C-C, aromatic C-O, and C-O-C stretching vibrations, respectively [55]. After curcumin encapsulation, the peaks of PS-0-C, PS-2-C, PS-4-C, PS-6-C, and PS-8-C at 1602 cm^−1^ shifted to 1636 cm^−1^, and the peak ranges were broadened due to potential interactions between components within the particles. The characteristic absorbance peak near 3520 cm^−1^ corresponding to the -OH stretching vibration of curcumin also disappeared after encapsulation; these changes confirm that curcumin is well encapsulated by PS-0, PS-2, PS-4, PS-6, and PS-8 [56,57]. Similar phenomena have been observed in studies on native banana starch nanoparticles or modified banana starch nanoparticles encapsulating curcumin [58]. According to Acevedo-Guevara, the interaction between curcumin and porous starch occurs through hydrogen bonding between curcumin and the hydroxyl groups of various glucose units.

#### 3.3.5. Thermal Stability Analysis

**Figure 8:**
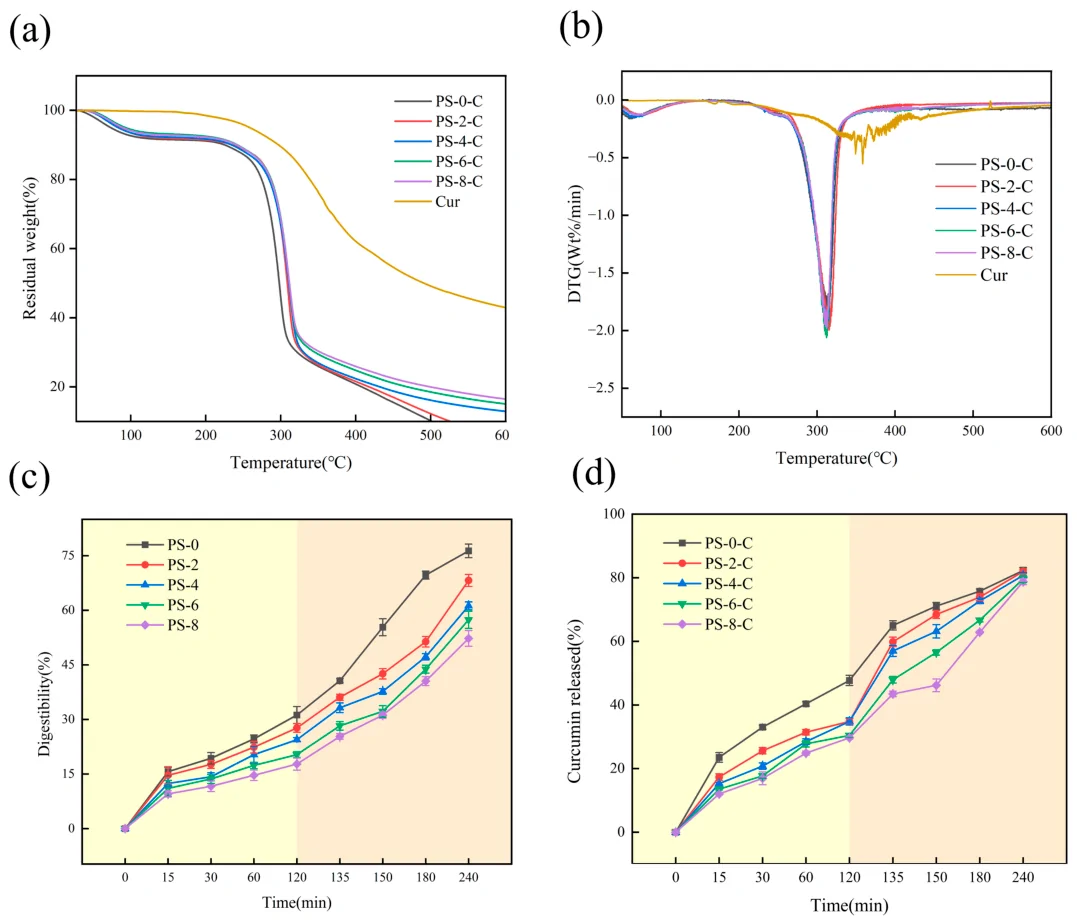
TG (**a**) and DTG (**b**) curves of the porous starch–curcumin microspheres; digestibility of porous starches prepared at different enzymatic hydrolysis times (**c**) and curcumin release rate from the porous starch−curcumin microspheres after digestion (**d**). In the background colors of the chart, yellow represents the gastric digestion stage, and orange represents the intestinal digestion stage. Error bars are standard deviations.

The TG and DTG results for curcumin and the porous starch–curcumin microspheres are shown in Figure 8a. The first weight loss stage occurs at around 100 °C and is mainly due to the evaporation of moisture from the starch. The second stage occurs between 150 and 360 °C and is attributed to the degradation of starch and curcumin. In contrast, free curcumin begins to lose mass at around 188 °C and undergoes only one weight loss stage. After curcumin encapsulation, the characteristic weight loss of curcumin is no longer observed in the TG curves of the porous starch–curcumin microspheres, leaving only the degradation stage of starch, which indicates that curcumin forms a curcumin-loaded porous starch with the porous starch rather than a simple physical mixture [40].

As shown in Figure 6b, the final residual weights of the different curcumin-loaded starch samples do not differ substantially. However, in the second weight loss stage, the mass loss of free curcumin starts at around 188 °C, whereas the thermal degradation of the porous starch–curcumin samples occurs at around 250 °C; the incorporation of carrier materials may delay the degradation of loaded curcumin [40].

#### 3.3.6. In Vitro-Simulated Digestion Analysis

As shown in Figure 8c, during simulated gastric and intestinal digestion, the digestibility of all starch samples increases more slowly in the gastric phase than in the intestinal phase. After complete digestion, the digestibility of native chickpea starch reaches 76.33%. For the porous starches prepared with different apertures, the larger the pore size, the stronger the resistance to digestion and the higher the resistant starch content. Figure 5a shows the proportions of rapidly digestible starch (RDS), slowly digestible starch (SDS), and resistant starch (RS) of the starches prepared at different enzymatic hydrolysis times. The first 20 min of starch digestion is RDS, and the division between SDS and RS occurs at 120 min of digestion [59].

As shown in Figure 8d, during the simulated gastric digestion phase (0–120 min), curcumin was released slowly from all samples. At 120 min, the cumulative release of the control sample PS-0-C had reached approximately 39%, whereas the porous starch samples released only 25–34%, with the release rate decreasing progressively with prolonged enzymatic hydrolysis time, and PS-8-C exhibited the lowest release (approximately 25%). These results indicate that the porous structure formed by enzymatic hydrolysis exerts a pronounced controlled release on curcumin. On the one hand, enzymatic hydrolysis generates numerous pores on the surface and in the interior of the starch granules, so that curcumin is physically adsorbed and loaded within the internal pore channels rather than merely attached to the granule surface; the physical barrier provided by the starch matrix thus hinders the outward diffusion of curcumin into the gastric fluid [60]. On the other hand, curcumin itself is extremely poorly water-soluble and, although relatively stable under acidic conditions, is difficult to dissolve, which is one of the main reasons for its low oral bioavailability. As the hydrolysis time increases, the number, diameter, and depth of the pores increase, more adsorption sites become distributed inside the granules, and the diffusion path of curcumin becomes longer, resulting in a more pronounced sustained-release effect [8].

During the simulated intestinal digestion phase (120–240 min), the release rate of curcumin increased markedly in all groups upon entering the simulated intestinal fluid. This is mainly attributed to the rapid hydrolysis of the starch skeleton by pancreatic α-amylase, which causes the gradual collapse of the porous structure and the sustained release of curcumin originally embedded within the pores as starch digestion proceeds [60]. The greatest difference among the groups was observed at 135 min (approximately 59% for PS-0-C, 50% for PS-2-C, and only 39% for PS-8-C); thereafter, as the starch skeleton continued to degrade, the differences among the groups gradually narrowed, and by 240 min the cumulative release of all samples had converged to approximately 80%. This indicates that the larger pore size and pore volume generated by prolonged enzymatic hydrolysis retard the release rate of curcumin without affecting its final cumulative release, thereby achieving a sustained-release effect while ensuring the liberation of curcumin.

## 4. Conclusions

This study successfully prepared chickpea porous starch through the synergistic action of α-amylase and glucoamylase and systematically evaluated its performance as a natural delivery carrier for curcumin. With increasing enzymatic hydrolysis time, dense micropores formed on the granule surface, and the specific surface area and pore volume increased significantly, while the original C-type crystalline structure was well retained. The porosification treatment markedly improved the solubility, swelling power, water- and oil-holding capacities, transparency, and freeze–thaw stability of the starch, while reducing its pasting viscosity. Among the prepared samples, porous starch hydrolyzed for 8 h (PS-8) exhibited the strongest adsorption performance, achieving a curcumin encapsulation efficiency of up to 80.65%. FTIR and thermogravimetric analyses indicated that curcumin was loaded into the porous starch through hydrogen-bonding interactions, forming complexes that significantly enhanced the thermal stability and retention rate of curcumin while preserving its excellent antioxidant activity (DPPH and ABTS radical scavenging abilities). In vitro-simulated digestion further demonstrated that the porous structure enhanced the resistance of the starch to digestion and enabled the sustained release of curcumin. It should be noted that the differences among the samples arise from the combined effects of the enzymatic hydrolysis time, including the resulting pore structure and the concurrent changes in molecular composition and crystallinity. Overall, chickpea porous starch is a potential edible food-grade starch-based carrier, and this work provides a theoretical basis and practical reference for the development of legume-starch-based delivery systems and the efficient encapsulation of hydrophobic bioactive compounds.

## Figures and Tables

**Table 1:** Nitrogen adsorption parameters of porous starch.

Samples	BET (m^2/^g)	Pore Volume (cm^3^/g)	Average Pore Size (nm)
PS-0	0.156	0.001	12.319
PS-2	0.306	0.017	59.034
PS-4	0.328	0.019	153.880
PS-6	0.344	0.023	159.481
PS-8	0.367	0.038	169.618

Operation error: BET ± 0.002 (m^2^/g), pore volume ± 0.0002 (cm^3^/g), average pore size ± 0.002 (nm).

**Table 2:** Pasting performance parameters of porous starch.

Samples	PV (mPa·s)	TV (mPa·s)	BD (mPa·s)
PS-0	1131.25 ± 89.73 ^a^	901.75 ± 70.34 ^a^	229.50 ± 26.85 ^a^
PS-2	1000.83 ± 56.57 ^a^	839.67 ± 43.84 ^b^	161.16 ± 12.73 ^b^
PS-4	991.50 ± 70.41 ^b^	831.00 ± 49.90 ^b^	160.50 ± 20.51 ^b^
PS-6	981.00 ± 32.53 ^b^	818.50 ± 18.03 ^b^	162.50 ± 25.66 ^b^
PS-8	842.50 ± 82.29 ^c^	715.50 ± 59.26 ^c^	127.00 ± 15.17 ^c^
Samples	FV (mPa·s)	SB (mPa·s)	PaT (°C)
PS-0	1373.00 ± 105.57 ^a^	471.25 ± 41.69 ^a^	76.35 ± 0.41 ^c^
PS-2	1024.50 ± 67.18 ^a^	113.50 ± 23.33 ^b^	82.90 ± 1.73 ^b^
PS-4	936.50 ± 18.08 ^b^	79.50 ± 31.81 ^c^	88.43 ± 2.04 ^a^
PS-6	901.67 ± 18.74 ^c^	74.00 ± 0.71 ^c^	88.55 ± 1.72 ^a^
PS-8	788.00 ± 59.02 ^d^	72.50 ± 2.18 ^c^	90.35 ± 0.87 ^a^

PV represents peak viscosity, TV represents trough viscosity, BD is breakdown viscosity, FV represents final viscosity, SB is setback viscosity, and PaT represents pasting temperature. Results are expressed as mean ± standard deviation. Values in the same column followed by different superscript letters differ significantly (*p* < 0.05).

## Data Availability

The data supporting the conclusions of this article will be made available by the authors on request.
